# Impact of induction regimens on stem cell mobilization yields in newly diagnosed multiple myeloma

**DOI:** 10.1007/s00277-025-06372-4

**Published:** 2025-04-25

**Authors:** Soyean Kwon, Hye Yeon Park, Ja Min Byun, Dong-Yeop Shin, Youngil Koh, Junshik Hong, Inho Kim, Sung-Soo Yoon

**Affiliations:** 1 Department of Internal Medicine, Seoul National University Hospital, 101 Daehak-ro, Jongno-gu, Seoul, 03080 Republic of Korea; 2 Department of Translational Medicine, Seoul National University College of Medicine, Seoul, Republic of Korea

**Keywords:** Autologous stem cell transplantation (ASCT), Multiple myeloma (MM), Induction regimens, Stem cell mobilization, CD34 + cell yield, Daratumumab-VTD (DVTD)

## Abstract

Autologous stem cell transplantation (ASCT) is integral to treating newly diagnosed multiple myeloma (MM). While novel therapies improve response rates, they also hinder stem cell mobilization. This study evaluates the impacts of induction regimens on mobilization, collection, and ASCT outcomes. We analyzed 228 patients divided into three groups: bortezomib-thalidomide-dexamethasone (VTD, *N* = 117); bortezomib-lenalidomide-dexamethasone (VRD, *N* = 57); and daratumumab-VTD (DVTD, *N* = 54). Baseline characteristics showed no significant differences among the groups. Chemo-mobilization was most common in VTD (20.5%) compared to VRD (12.3%) and DVTD (5.6%). Total CD34 + cell yield (x10⁶/kg) was highest in VTD (7.1 ± 3.5) compared to VRD (5.8 ± 3.2) and DVTD (5.4 ± 2.4) [*p* = 0.0001]. Second mobilization was required most frequently in VRD (40.4%) compared to DVTD (24.1%) and VTD (16.2%) [*p* = 0.0010]. Plerixafor use was highest in VRD (40.4%) compared to DVTD (24.1%) and VTD (12.0%) [*p* = 0.0001]. Mobilization duration was longest in VRD (4.0 ± 1.9 days) and shortest in VTD (3.2 ± 1.7 days) [*p* = 0.0038]. Infused CD34 cells and platelet engraftment times were comparable among groups. Neutrophil engraftment was delayed in VRD (12.1 ± 0.9 days) compared to DVTD (11.8 ± 1.2) and VTD (11.6 ± 0.7) [*p* = 0.0014]. Prompt stem cell collection is essential in lenalidomide regimens to minimize mobilization challenges. While DVTD demonstrated comparable mobilization efficiency, it produced fewer CD34 cells than VTD, indicating potential challenges.

## Introduction

Multiple myeloma (MM) is a hematologic malignancy marked by the clonal expansion of plasma cells within the bone marrow, resulting in clinical manifestations such as anemia, osteolytic bone lesions, renal dysfunction, and hypercalcemia. MM constitutes approximately 10% of all hematologic cancers, with its incidence steadily increasing worldwide [1].

Although MM remains incurable, survival has significantly improved over the past decade, largely due to advancements in treatment options [2, 3]. Even with novel therapeutic agents in place, autologous stem cell transplantation (ASCT) remains an integral part of the treatment for newly diagnosed MM (NDMM) patients. Recent findings from the DETERMINATION trial reaffirmed the benefit of ASCT in VRD (bortezomib-lenalidomide-dexamethasone) treated patients, demonstrating improved progression-free survival (PFS) [4].

The success of ASCT depends on the ability to collect sufficient number of hematopoietic stem cells (HSCs). It is well established that to ensure engraftment, a minimum infusion of 2.0–2.5 × 10^6 stem cells per kilogram of body weight is required [5–8]. Since the introduction of peripheral blood stem cell transplantation (PBSCT), stem cells are now predominantly collected through peripheral blood [9]. Therefore the process of mobilization, which involves directing cells from the bone marrow into peripheral blood, has become the mainstay of adequate cell collection. This process can be affected by multiple variables, primarily by the induction regimen given prior to mobilization [10–12].

The most commonly used incumbent induction regimens include lenalidomide-based triplet or anti-CD38 antibody-based quadruplet therapies [13]. These regimens improved the depth of response but seem to have varying impacts on stem cell mobilization. Namely, lenalidomide has been associated with challenges in stem cell mobilization, particularly when used for extended periods [11]. It has been reported that daratumumab, similar to lenalidomide, negatively impacts stem cell yield and is associated with increased plerixafor use [14]. Given the evolving landscape of induction therapies and their impact on stem cell mobilization, it becomes essential to understand how different regimens affect mobilization yields in patients with NDMM.

In Korea, VRD regimen is commonly used as a first-line treatment for NDMM. DVTD regimen (daratumumab, bortezomib, thalidomide, dexamethasone) is also being used, though it is not covered by public insurance. This study aims to compare the mobilization outcomes according to induction regimens, specifically focusing on the impact of lenalidomide and daratumumab on stem cell yield reduction compared to the more traditional VTD regimen (bortezomib, thalidomide, dexamethasone). Additionally, we evaluated whether variations in stem cell yield translate into differences in ASCT-related metrics, such as engraftment times and survival outcomes, thereby providing a more comprehensive view of our cohort.

## Methods

### Study design and population

**Fig. 1:**
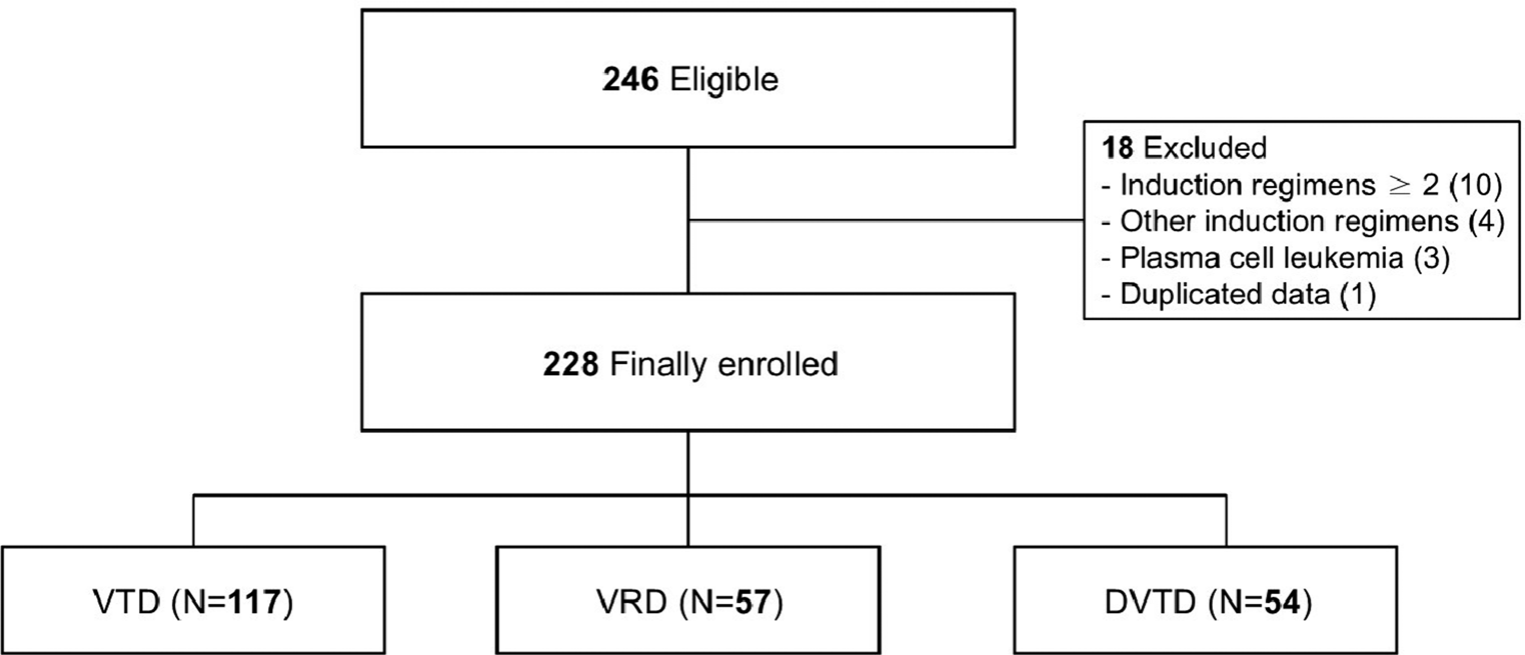
Study flow chart. VTD, bortezomib-thalidomide-dexamethasone; VRD, bortezomib-lenalidomide-dexamethasone; DVTD, daratumumab-bortezomib-thalidomide-dexamethasone

This retrospective cohort study focused on patients with MM undergoing ASCT at Seoul National University Hospital (SNUH) between January 2020 and December 2023. Patients aged 19 years or older who received VTD, VRD or DVTD as induction therapy prior to transplantation were eligible for inclusion. Patients treated with induction therapies other than these three regimens or who failed their initial induction therapy and required more than two types of induction therapies before ASCT were excluded. Patients diagnosed with plasma cell leukemia were also excluded from the study (Fig. 1).

Patients’ sex, International Staging System (ISS) stage, Revised International Staging System (RISS) stage, induction chemotherapy cycles, and treatment responses were reviewed. Details of the mobilization process were examined, along with the mobilization methods used, and the corresponding collection amounts and durations. Patients received subcutaneous granulocyte colony-stimulating factor (G-CSF) at a dose of 10 µg/kg for four days prior to the collection of CD34 + cells. For chemo-mobilization, cyclophosphamide and etoposide-based chemotherapy followed by G-CSF were administered before stem cell collection. Plerixafor was given in cases where standard mobilization methods, including a combination of chemotherapy and G-CSF or G-CSF alone, were unsuccessful in collecting at least 2.0 × 10^6^ CD34 + cells/kg over a two-day period. Additionally, it was preemptively administered during the collection period when mobilization failure was anticipated. This included situations where the peripheral blood CD34 + cell count was below 10 cells/µL and the collection on the first day yielded less than 0.7 × 10^6^ CD34 + cells/kg, or when the peripheral blood CD34 + cell count was below 15 cells/µL and the first-day collection produced less than 1.0 × 10^6^ CD34 + cells/kg.

The conditioning regimens used and the quantity of CD34 + cells infused during ASCT were also reviewed. The time to platelet recovery was defined as the interval from the day of ASCT to the first day on which the platelet count remained above 50,000/uL for at least three consecutive days. Neutrophil engraftment time was defined as the period from the day of ASCT to the first day on which the absolute neutrophil count (ANC) exceeded 500/uL for at least three consecutive days.

### Statistical analysis

The primary objective of this study is to evaluate whether there are differences in stem cell yield for ASCT based on the three induction regimens. Patient characteristics according to induction regimen were analyzed using the chi-square test for categorical variables and ANOVA for continuous variables with post hoc analysis. In cases where the expected frequency was less than 5, analysis was performed using Fisher’s exact test. Continuous variables that did not meet the assumption of normality were analyzed using the Kruskal-Wallis test. The p-values were adjusted using the Bonferroni method. Two-sided *p*-values of less than 0.05 were considered statistically significant.

To evaluate the impact of the induction regimen on stem cell yield, a multivariate linear regression analysis was performed with age, sex, chemotherapy response, first mobilization method, and plerixafor use as covariates. The optimal model was selected using a backward model selection process. A scatter plot was generated for the stem cell count per chemotherapy cycle for each induction regimen. Subsequently, a linear regression model was applied to visualize the data. A survival graph was created using Kaplan-Meier curves, and differences between the groups were tested using the log-rank test. Statistical analysis was performed with R version 4.4.1.

The study protocol was reviewed and approved by the institutional review board of SNUH (IRB number: H-2207-083-1339). This study was carried out in accordance with the recommendations of the Declaration of Helsinki for biomedical research involving human subjects.

## Results

### Patient baseline characteristics

Baseline characteristics according to induction regimen are summarized in Table 1. A total of 117 patients were included in the VTD (T) group, 57 in the VRD (R) group, and 54 in the DVTD (D) group. There were no significant differences among the groups in terms of age, sex distribution, or the distribution of ISS and R-ISS stages. The rates of achieving a very good partial response (VGPR) or better as the best response following induction chemotherapy were generally comparable across all three groups, although the DVTD group showed a tendency toward better outcomes. There was 1 patient in the VTD group and 2 patients in the VRD group whose best responses were lower than a partial response (PR). The number of chemotherapy cycles was determined by the clinician’s judgment, with fewer cycles for early responders and additional cycles for those requiring a deeper response or facing logistical constraints. The number of induction chemotherapy cycles before mobilization was higher in the VTD (3.8 ± 0.9) and VRD groups (3.5 ± 1.0) compared to the DVTD group (2.5 ± 0.7). (VTD vs. DVTD; *p* < 0.0001, VRD vs. DVTD; *p* < 0.0001).

**Table 1:** Baseline characteristics by induction regimen

Characteristics	VTD (*N* = 117)	DVTD (*N* = 54)	VRD (*N* = 57)	*p*-value			
				All	D vs. T	T vs. *R*	D vs. *R*
Age							
Mean (range)	60.7 ± 7.6	59.8 ± 7.4	59.4 ± 7.8	0.4140	0.5827	0.3260	1.0000
Sex							
Female	63 (53.8%)	29 (53.7%)	24 (42.1%)	0.3103	1.0000	0.5888	0.9053
Male	54 (46.2%)	25 (46.3%)	33 (57.9%)				
ISS							
1	44 (40.7%)	22 (42.3%)	21 (37.5%)	0.8658	1.0000	1.0000	1.0000
2	34 (31.5%)	16 (30.8%)	22 (39.3%)				
3	30 (27.8%)	14 (26.9%)	13 (23.2%)				
RISS							
1	21 (32.3%)	8 (21.1%)	9 (20.5%)	0.6152	1.0000	1.0000	1.0000
2	33 (50.8%)	22 (57.9%)	25 (56.8%)				
3	11 (16.9%)	8 (21.1%)	10 (22.7%)				
Chemotherapy cycles before mobilization							
Mean (range)	3.8 ± 0.9	2.5 ± 0.7	3.5 ± 1.0	< 0.0001	< 0.0001	0.1906	< 0.0001
Best response							
≥ VGPR	76 (65.0%)	45 (83.3%)	42 (73.7%)	0.0428	0.0688	0.9760	0.9467
sCR	1 (0.9%)	1 (1.9%)	6 (10.5%)				
CR	5 (4.3%)	7 (13.0%)	5 (8.8%)				
VGPR	70 (59.8%)	37 (68.5%)	31 (54.4%)				
PR	40 (34.2%)	9 (16.7%)	13 (22.8%)				
SD	1 (0.9%)	0 (0.0%)	2 (3.5%)				

VTD, bortezomib-thalidomide-dexamethasone; VRD, bortezomib-lenalidomide-dexamethasone; DVTD, daratumumab-VTD

T, VTD; R, VRD; D, DVTD

ISS, International Staging System; RISS, Revised International Staging System

VGPR, very good partial response; sCR, stringent complete remission; PR, partial response; SD, stable disease

### Mobilization methods and yields

The details regarding the mobilization methods and yields for the three groups are summarized in Table 2. The proportion of patients undergoing first mobilization through chemotherapy was statistically higher in VTD, compared to DVTD group. (VTD vs. DVTD; 20.5% vs. 5.6%, *p* = 0.038). In the VTD group, 22 patients (18.8%) underwent chemo-mobilization with cyclophosphamide during the first mobilization, and 2 patients (1.7%) used DCEP. In the VRD group, 6 patients (10.5%) used cyclophosphamide, and 1 patient (1.7%) used the DCEP regimen. In the DVTD group, 3 patients (5.6%) used cyclophosphamide.

**Table 2:** Mobilization outcomes by induction regimen

Characteristics	VTD (*N* = 117)	DVTD (*N* = 54)	VRD (*N* = 57)	*p*-value			
				All	D vs. T	T vs. *R*	D vs. *R*
First mobilization method							
G-CSF mobilization	93 (79.5%)	51 (94.4%)	50 (87.7%)	0.0312	0.0380	0.6327	0.9675
Chemo-mobilization	24 (20.5%)	3 (5.6%)	7 (12.3%)				
Cyclophosphamide	22 (18.8%)	3 (5.6%)	6 (10.5%)				
DCEP	2 (1.7%)	0 (0.0%)	1 (1.7%)				
Second mobilization method							
Done	19 (16.2%)	13 (24.1%)	24 (42.1%)	0.0010	0.1113	0.0011	0.2778
Plerixafor mobilization	13 (11.1%)	13 (24.1%)	22 (38.5%)				
Preemptive	9 (7.6%)	4 (7.4%)	5 (8.7%)				
Chemo-mobilization	6 (5.1%)	0 (0.0%)	2 (3.5%)				
Cyclophosphamide	6 (5.1%)	0 (0.0%)	0 (0.0%)				
Cytarabine	0 (0.0%)	0 (0.0%)	2 (3.5%)				
Not done	98 (83.8%)	41 (75.9%)	33 (57.9%)				
Third mobilization method							
Plerixafor mobilization	1 (0.9%)	0 (0.0%)	1 (1.8%)	0.6117	1.0000	1.0000	1.0000
Total CD34 + cell count							
Mean, 10^6/kg (range)	7.1 ± 3.5	5.4 ± 2.4	5.8 ± 3.2	0.0001	0.0003	0.0028	0.8523
CD34 + cell count per day (Total)							
Mean, 10^6/kg (range)	3.0 ± 2.7	2.2 ± 2.2	2.5 ± 2.9	0.0010	0.0029	0.0054	1.0000
First mobilization CD34 + cell count							
Mean, 10^6/kg (range)	6.4 ± 3.8	4.5 ± 2.9	4.1 ± 3.5	< 0.0001	0.0006	< 0.0001	0.8051
Total mobilization duration							
Mean, days (range)	3.2 ± 1.7	3.5 ± 1.5	4.0 ± 1.9	0.0038	0.1327	0.0017	0.2912
Plerixafor use	14 (12.0%)	13 (24.1%)	23 (40.4%)	0.0001	0.2190	0.0001	0.3104

G-CSF, Granulocyte-Colony stimulating factor; DCEP, dexamethasone-cyclophosphamide etoposide-cisplatin

The total CD34 + cell yield [10^6/kg] was highest in the VTD group (7.1 ± 3.5), compared to the VRD group (5.8 ± 3.2) and the DVTD group (5.4 ± 2.4). (VTD vs. VRD; *p* = 0.0028, VTD vs. DVTD; *p* = 0.0003) The CD34 + cell collection per day [10^6/kg] was higher in the VTD group (3.0 ± 2.7), compared to the VRD group (2.5 ± 2.9) and the DVTD group (2.2 ± 2.2). (VTD vs. DVTD; *p* = 0.0029, VTD vs. VRD; *p* = 0.0054) The CD34 + cell yield obtained from the first mobilization [10^6/kg] was also significantly higher in the VTD group (6.4 ± 3.8), compared to the VRD group (4.1 ± 3.5) and the DVTD group (4.5 ± 2.9). (VTD vs. DVTD; *p* = 0.0006, VTD vs. VRD; *p* < 0.0001).

### Factors affecting mobilization yield

To explore prognostic factors affecting mobilization yield, a multivariate linear regression analysis was conducted, adjusting for other variables. The induction regimen was identified as a significant factor for total CD34 + cell yield, with DVTD and VRD showing significantly lower CD34 + cell counts compared to VTD (VTD vs. DVTD, *p* = 0.0065; VTD vs. VRD, *p* = 0.0140). Age at diagnosis and the first mobilization method were also factors that influenced the total CD34 + cell count as shown in Table 3.

**Table 3:** Variables affecting total CD34 + cell counts

Characteristics	Status	Estimates	Standard Error	T value	*P* value
Intercept		9.038	1.835	4.925	< 0.0001
Age	Per 1 year	-0.083	0.026	-3.149	0.0018
First mobilization method	G-CSF (Intercept)	-	-	-	-
	Chemotherapy	2.566	0.568	4.513	< 0.0001
Induction regimen	VTD (Intercept)	-	-	-	-
	DVTD	-1.373	0.500	-2.745	0.0065
	VRD	-1.205	0.487	-2.474	0.0140

**Fig. 2:**
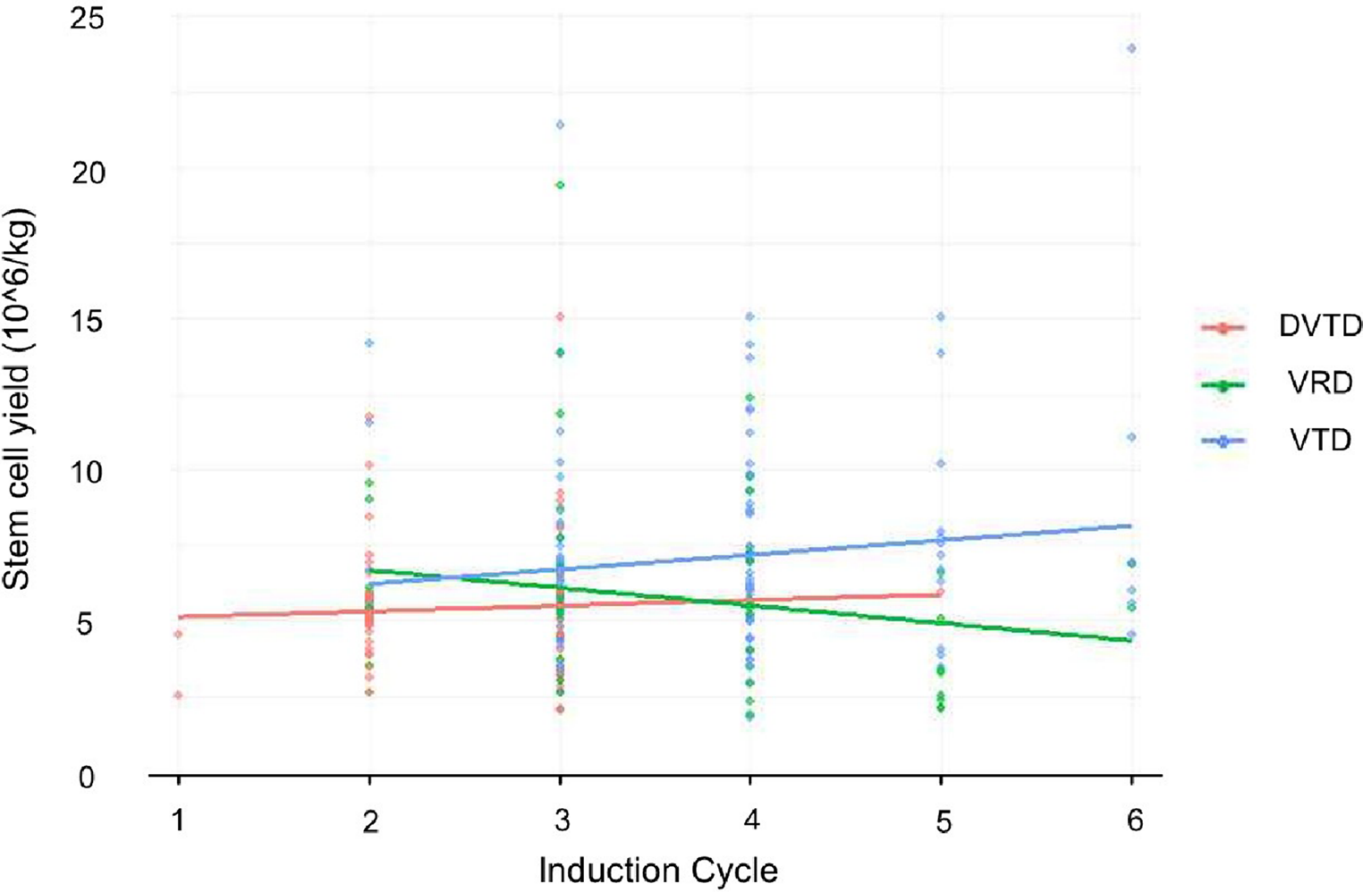
Stem cell yield across induction cycles. 10 ^6/kg, yield per kilogram of body weight

**Fig. 3:**
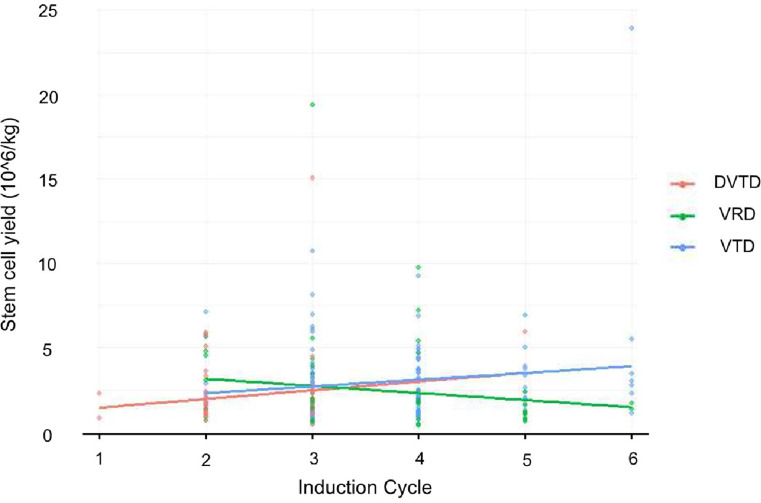
Mean stem cell yield per induction cycle. 10^6/kg, yield per kilogram of body weight

If the induction regimen significantly affects stem cell yield, this effect is expected to become more evident with an increasing number of chemotherapy cycles before ASCT, and this relationship can be represented by linear regression. Thus, a linear regression model was created for each group, with chemotherapy cycles on the x-axis and total stem cell yield on the y-axis. In the VRD group, each additional chemotherapy cycle was linked to a decrease of 0.5846 × 10^6/kg, while in the DVTD group, it corresponded to an increase of 0.1843 × 10^6/kg, and in the VTD group, an increase of 0.4814 × 10^6/kg (Fig. 2). For stem cell yield per day, each additional cycle in the VRD group showed a decrease of 0.4206 × 10^6/kg, while the DVTD and VTD groups showed increases of 0.1638 and 0.4314, respectively (Fig. 3). In all models, only the VRD group showed a trend of decreasing stem cell yield with more chemotherapy cycles.

### ASCT outcomes

The ASCT related metrics are shown in Table 4. The amount of infused CD34 + cells [10^6/kg] was comparable among the three groups. (VTD, VRD, DVTD; 4.1 ± 1.4, 3.8 ± 1.3, 3.8 ± 1.3, *p* = 0.2542) There was no significant difference in the time to platelet engraftment [days] among the three groups (VTD, VRD, DVTD; 15.1 ± 3.4, 15.6 ± 4.2, 15.7 ± 2.6, *p* = 0.1751). The time to neutrophil engraftment was longest in the VRD group (12.1 ± 0.9), compared to the VTD group (11.6 ± 0.7) and DVTD group (11.8 ± 1.2). (VRD vs. VTD; *p* < 0.0001, VRD vs. DVTD; *p* = 0.0164).

**Table 4:** ASCT outcomes by induction regimen

Characteristics	VTD (*N* = 117)	DVTD (*N* = 54)	VRD (*N* = 57)	*p*-value			
				All	D vs. T	T vs. *R*	D vs. *R*
Infused CD34 + cell count							
Mean, 10^6/kg (range)	4.1 ± 1.4	3.8 ± 1.3	3.8 ± 1.3	0.2542	0.2348	0.3084	1.0000
Time to neutrophil engraftment							
Mean, days (range)	11.6 ± 0.7	11.8 ± 1.2	12.1 ± 0.9	0.0014	0.3447	< 0.0001	0.0164
Time to platelet engraftment							
Mean, days (range)	15.1 ± 3.4	15.7 ± 2.6	15.6 ± 4.2	0.1751	0.0948	0.6586	0.5112
Progression-free survival							
Median (months)	37	NR	26	0.1287	0.3018	1.0000	0.1446
Overall survival							
Median (months)	NR	NR	NR	0.0152	0.0720	0.1419	1.0000

ASCT, autologous stem cell transplantation

After ASCT, maintenance therapy was administered in 41 patients (35.0%) in the VTD group, 42 (77.8%) in the DVTD group, and 40 (70.2%) in the VRD group (*p* < 0.001). One patient in the VTD group and one in the VRD group received elranatamab maintenance, while two patients in the DVTD group received thalidomide; all others received lenalidomide. The Kaplan-Meier curves for progression-free survival (PFS) and overall survival (OS) among the three groups are shown in Supplementary Figs. 1 and 2. The median values of survival outcomes are presented in Table 4. Although not statistically significant, the DVTD group showed a trend toward better PFS and OS outcomes.

## Discussion

Our study investigated stem cell mobilization outcomes following induction therapy with VTD, VRD, and DVTD regimens in patients with NDMM, revealing several important findings that contribute to optimizing treatment strategies.

Early stem cell collection is widely recognized as crucial for patients receiving VRD [15–17]. Prolonged use of lenalidomide, a key component of VRD, leads to cumulative hematologic toxicity—especially myelosuppression [16]. In this study, we used linear regression not only to quantify how extended VRD therapy reduces CD34 + cell yield but also to identify the optimal window for stem cell collection. Specifically, each additional cycle of VRD was associated with a significant decline of 0.5846 × 10^6/kg in total CD34 + cell yield. Considering that all patients in the VRD group who achieved a PR or better exhibited this response by the end of cycle 3, we recommend attempting stem cell collection within 3 cycles as soon as a response is confirmed, to minimize any potential impact on stem cell yield. Furthermore, the time to neutrophil engraftment was significantly longer in the VRD group than in the VTD group, raising concerns about the quality of engrafted stem cells in patients receiving VRD therapy.

Our data demonstrated results favoring the VTD regimen when comparing stem cell yield outcomes, the rate of second mobilization, plerixafor use, and mobilization duration. This may be attributed to the significantly higher proportion of chemo-mobilization in the VTD group as the first mobilization method. Although our analysis focused solely on total CD34 + cell count, the induction regimen remained a significant variable even after adjusting for the first mobilization method. Furthermore, despite the VRD group having the second-highest rate of chemo-mobilization, its mobilization duration, need for second mobilization, and plerixafor use were less favorable than those in the DVTD group, further emphasizing the impact of the induction regimen.

Second, our findings revealed that the CD34 + cell yield was significantly lower in the DVTD group compared to the VTD group, likely due to the effects of daratumumab. However, the DVTD regimen demonstrates clear advantages over VTD. While the VTD group required a higher rate of chemo-mobilization to compensate for suboptimal induction responses in tumor control, the DVTD regimen follows a fixed four-cycle induction protocol, leading to fewer cycles before stem cell collection, as shown in Table 1. Moreover, this shift reflects a meaningful improvement in clinical practice, reducing the reliance on aggressive tumor control measures. Importantly, the reduced mobilization efficiency in the DVTD group had minimal clinical impact, as sufficient stem cell collection was successfully achieved in both groups without the need for prolonged mobilization or additional interventions. Notably, although not statistically significant, the DVTD group showed a trend toward better survival outcomes in our cohort, though this may be influenced by the higher maintenance therapy rate.

Our linear regression analysis showed that in both VTD and DVTD, additional chemotherapy cycles modestly increased total stem cell yield (Fig. 2). However, this trend should be interpreted with caution, as the data distribution suggests potential bias. In VTD, a few outliers with high stem cell yield were observed at higher cycle numbers, while in DVTD, most data points were concentrated below three cycles, with only one case at five cycles exceeding three cycles. This skewed distribution may have influenced the regression slope, and therefore, we caution against concluding that mobilization yield consistently increases with additional induction cycles.

This study has limitations inherent to retrospective data, including inevitable biases due to missing key variables such as ISS and R-ISS stages, which were excluded from the multivariate regression model. And Dara-VRD (daratumumab + VRD) is emerging as a modern quadruplet regimen with promising efficacy in NDMM, though not widely utilized in this cohort due to insurance constraints and timing. Future studies with larger patient cohorts including Dara-VRD data are warranted to validate these findings and strengthen the robustness of the conclusions and to offer further insights into quadruplet-induced mobilization challenges.

In conclusion, our study emphasizes the importance of timely stem cell collection in VRD-treated patients to overcome mobilization challenges and highlights the balanced performance of DVTD, which achieves sufficient mobilization outcomes with additional clinical benefits. These findings support the continued use of VTD in resource-restrained settings, given its efficacy and feasibility for ASCT, and contribute to more personalized treatment approaches for NDMM patients undergoing ASCT.

## Data Availability

No datasets were generated or analysed during the current study.
